# Ulcerative Colitis-associated *E. coli* pathobionts potentiate colitis in susceptible hosts

**DOI:** 10.1080/19490976.2020.1847976

**Published:** 2020-12-01

**Authors:** Hyungjun Yang, Hengameh Chloé Mirsepasi-Lauridsen, Carsten Struve, Joannie M. Allaire, Adeline Sivignon, Wayne Vogl, Else S. Bosman, Caixia Ma, Abbas Fotovati, Gregor S. Reid, Xiaoxia Li, Andreas Munk Petersen, Sébastien G. Gouin, Nicolas Barnich, Kevan Jacobson, Hong Bing Yu, Karen Angeliki Krogfelt, Bruce A. Vallance

**Affiliations:** a Department of Pediatrics, BC Children’s Hospital, University of British Columbia, Vancouver, BC, Canada; b Department of Bacteria, Parasites and Fungi, Statens Serum Institute, Copenhagen, Denmark; c Department of Biology, University of Copenhagen, Copenhagen, Denmark; d Université Clermont Auvergne, Laboratoire Microbes Intestin Inflammation Et Susceptibilité De l’Hôte (M2ish), Inserm U1071, M2iSH, F-63000, Clermont-Ferrand, France; e INRA, Unité Sous Contrat 2018, Clermont-Ferrand, France; f Department of Cellular and Physiological Sciences, University of British Columbia, Vancouver, BC, Canada; g Department of Immunology, Cleveland Clinic Lerner Research Institute, Cleveland, OH, USA; h Department of Gastroenterology, Copenhagen University Hospital, Hvidovre, Denmark; i Department of Clinical Microbiology, Copenhagen University Hospital, Hvidovre, Denmark; j Université De Nantes, Chimie Et Interdisciplinarité, Synthèse, Analyse, Modélisation (CEISAM), UMR CNRS 6230, UFR Des Sciences Et Des Techniques, Nantes, France; k Lead Contact

**Keywords:** Inflammatory bowel disease, Ulcerative Colitis, Crohn’s disease, intestinal microbiota, in vivo mouse model

## Abstract

Ulcerative colitis (UC) is a chronic inflammatory condition linked to intestinal microbial dysbiosis, including the expansion of *E. coli* strains related to extra-intestinal pathogenic *E. coli*. These “pathobionts” exhibit pathogenic properties, but their potential to promote UC is unclear due to the lack of relevant animal models. Here, we established a mouse model using a representative UC pathobiont strain (p19A), and mice lacking single immunoglobulin and toll-interleukin 1 receptor domain (SIGIRR), a deficiency increasing susceptibility to gut infections. Strain p19A was found to adhere to the cecal mucosa of *Sigirr* -/- mice, causing modest inflammation. Moreover, it dramatically worsened dextran sodium sulfate-induced colitis. This potentiation was attenuated using a p19A strain lacking α-hemolysin genes, or when we targeted pathobiont adherence using a p19A strain lacking the adhesin FimH, or following treatment with FimH antagonists. Thus, UC pathobionts adhere to the intestinal mucosa, and worsen the course of colitis in susceptible hosts.

## Introduction

Inflammatory bowel diseases (IBD) are chronic inflammatory conditions traditionally divided into Crohn’s disease (CD) and Ulcerative Colitis (UC). CD is a chronic, segmentally localized and penetrating granulomatous inflammatory disease that can affect any part of the gastrointestinal (GI) tract^1^. In contrast, UC is a relapsing, superficial mucosal inflammatory disease restricted to the colon that is characterized by bloody diarrhea during flares of the disease. The etiology of IBD is still unknown, but thought to reflect the convergence of environmental stimuli and genetic susceptibility factors that together promote chronic intestinal inflammation. A variety of IBD susceptibility genes have been identified, many of them encoding proteins that regulate innate immune control over enteric bacterial pathogens, such as NOD2, NALP3 and ATG16L1^2^. Correspondingly, numerous investigations suggest the characteristic intestinal inflammation seen in IBD patients occurs concurrently with, and is potentially exacerbated by dysbiotic changes in the intestinal microbiome^3^. In many studies, the loss of potentially beneficial microbes such as *Faecalbacterium prausnitzii* and *Lactobacilli* species, as well as a reduction in microbial diversity have been suggested as pathogenic steps toward the development of IBD.

Even so, the loss of beneficial microbes alone does not explain the complicated role played by the gut microbiota in IBD. For example, placebo-controlled studies have shown that antibiotic treatment can induce remission in some IBD patients^4,5^. Moreover, the overgrowth of specific *Escherichia coli* species has been suspected since the 1970’s as the reason for relapses in some patients with IBD^6^. In 1988, Burke et al ^7^ showed a significantly higher frequency of adherent *E. coli* isolated from UC and CD patients undergoing disease relapse, as compared to healthy persons. Several additional studies have demonstrated an increased prevalence of *E. coli* with virulence properties in the GI tracts of IBD patients, especially within IBD patients undergoing disease relapse^8,9,10^, Recent studies indicate the involvement of adherent-invasive *E. coli* (AIEC) in the pathogenesis of CD^11,12,13,14,^ with demonstrations that AIEC can invade intestinal epithelial cells as well as persist within macrophages^15, 16^. The development of several mouse models of AIEC infection has aided in defining the virulence properties of AIEC, as well as the host factors that control susceptibility to these microbes^17, 18^.

Although AIEC is frequently seen in CD patients, it is uncommon in UC patients. Instead, diffusely adherent *E. coli* (DAEC) have been linked to UC^19^. Bacteriological analysis of biopsies and fecal samples from UC patients shows the increased prevalence of *E. coli* species belonging to the B2 phylogenetic group that harbor extra-intestinal pathogenic *E. coli* (ExPEC) genes^20, 21^, including α-hemolysin genes, as well as the gene encoding the adhesin FimH. Notably, studies focusing on a representative UC-associated *E. coli* strain termed p19A found that upon phagocytosis, this hemolysin-expressing microbe triggered the death of dendritic cells as well as strongly stimulated the secretion of the proinflammatory cytokines TNF-α, IL-6 and IL-23^22^. Studies of p19A interactions with the Caco-2 intestinal epithelial cell line have also shown that p19A induces α-hemolysin dependent damage to the tight junction protein occludin and thereby disrupts tight junctions in Caco-2 cells, increasing barrier permeability^20, 23^. Despite these pathogenic properties, the potential for these *E. coli* pathobionts to promote the course of UC remains unclear due to the lack of relevant animal models.

Recently, we have made significant progress developing mouse models of enteric infection, using mice deficient in Single IgG IL-1 Related Receptor (SIGIRR). SIGIRR is highly expressed by intestinal epithelial cells, acting as a negative regulator of innate signaling through most Toll-like receptors (TLR) as well as IL-1 R^24^. The absence of SIGIRR results in an increased inflammatory tone within the GI tract under baseline conditions, as well as exaggerated inflammatory and antimicrobial responses to murine and human enteric bacterial pathogens ^25, 26^. Similarly, many IBD patients, even in remission, have a higher inflammatory tone in their GI tracts. Interestingly, upon *Citrobacter rodentium* infection, *Sigirr* -/- mice exhibited exaggerated antimicrobial responses that were largely ineffective against this pathogen, but instead depleted competing resident commensal microbes. We also found *Sigirr* -/- mice to be uniquely susceptible to infection by the clinically important foodborne pathogen *Campylobacter jejuni*, developing overt gastroenteritis upon infection^26^.

Based on these findings, we explored the potential to establish a mouse model of GI infection by the UC-associated *E. coli* strain p19A, as well as characterize the pathogenic features of p19A. Initial studies showed that mice orally gavaged with high and/or repeated doses of p19A suffered severe damage within the small and large intestine as well as systemic spread of the bacteria, leading to high mortality rates ^27^. Interestingly, these responses were at least partially dependent on α-hemolysin. In our current study, to develop a mouse model more representative of UC, mice were pretreated with vancomycin and then infected with a single moderate dose of p19A. Indeed, vancomycin pretreatment of wildtype C57BL/6 mice enabled p19A to persistently colonize the intestinal lumen, whereas the same treatment of *Sigirr* **-/-** mice also led to p19A adherence to the cecal mucosal surface. While p19A infection caused modest cecal inflammation in non-DSS treated *Sigirr* -/- mice, it dramatically worsened the course of colitis in DSS treated *Sigirr* -/- mice, with p19A showing increased adherence to the inflamed intestinal mucosa. Notably, this adherence, and its ability to worsen colitis was lost with a p19A strain lacking the adhesin FimH, or when mice were given FimH antagonists to block p19A adhesion. Moreover, a p19A strain lacking α-hemolysin genes (∆*hlyI*∆*hlyII*) was attenuated in its ability to promote DSS colitis. Our findings thus provide evidence that UC associated *E. coli* strains can readily and persistently colonize the intestines of susceptible hosts, and significantly worsen the course of colitis in a manner dependent on specific virulence factors, including α-hemolysin and the type 1 fimbrial adhesin FimH.

## Results

### Vancomycin pretreatment facilitates persistent p19A intestinal colonization of C57BL/6 mice

**Figure 1.**
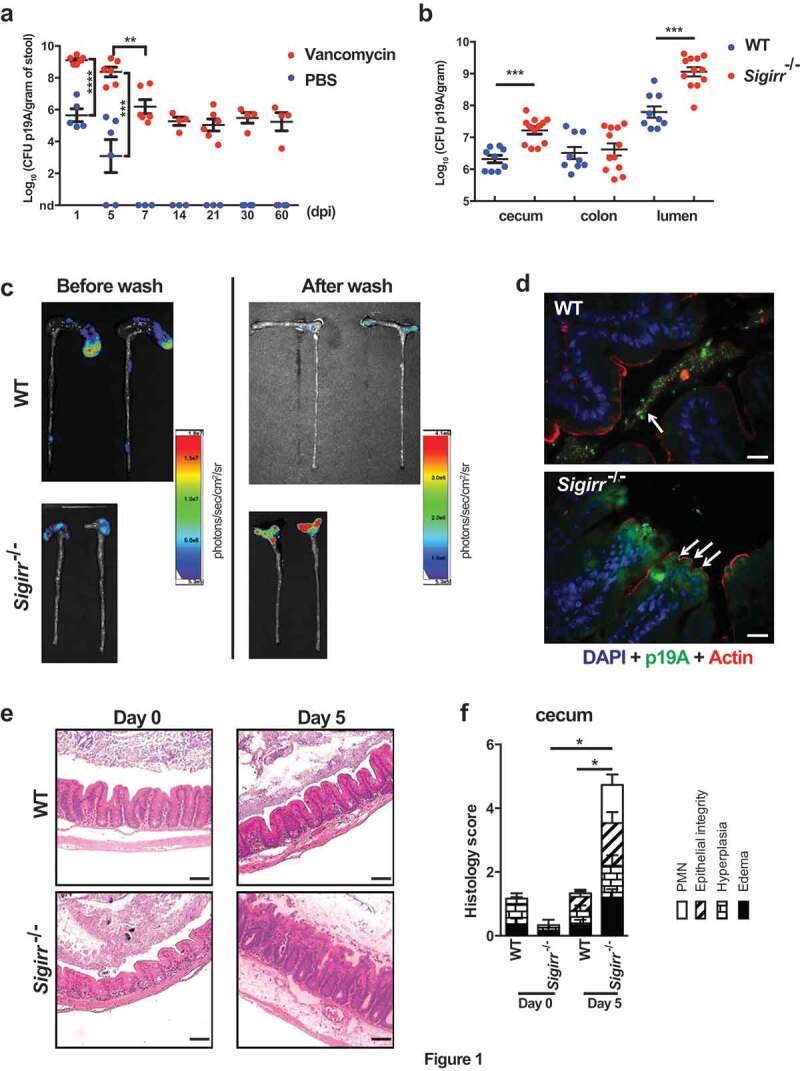
*Sigirr*-/- mice show increased susceptibility to p19A colonization of the GI tract

A variety of B2 phylogenetic group *E. coli* strains have been isolated from patients with either active or inactive UC^20^. Whether these *E. coli* strains play a pathogenic role in UC remains unclear. To address this, we examined the ability of p19A, one of the best-characterized human UC isolates ^23^, to colonize the intestines of mice. Groups of C57BL/6 wildtype (WT) mice were left untreated, or pretreated with vancomycin for 6 hours, followed by infection with p19A. We then quantified the number of p19A in the stool by plating. As shown in Figure 1a), mice not receiving vancomycin showed only moderate shedding of p19A in the stool (10^5^ colony forming units (CFU)/gram) at 1 day post-infection (dpi), but by 5 and 7 dpi, p19A was greatly reduced (~10^3^ CFU/gram) or even cleared from the stool. By 14 dpi, p19A was cleared from the stool of all mice. In contrast, pretreatment with vancomycin dramatically increased p19A shedding to 10^9^ CFU/gram of stool at 1 dpi, and similar levels (5 x 10^8^ CFU/gram) were shed at 5 dpi. The shedding of p19A gradually decreased to an average of 10^6^ CFU/gram by 7 dpi and to 10^5^ CFU/gram of stool by 14 dpi. This level of shedding continued out to 60 dpi, and potentially beyond, showing that p19A can persistently colonize the intestines of mice following antibiotic treatment.

(**a**) The numbers of p19A bacteria in feces from PBS- (n = 3–6) or vancomycin- (n = 4–6) pretreated (6 h before infection) WT (C57BL/6) mice were determined over a 60-day infection period. nd, not detected. Data are representative of two independent experiments. (**b**) The numbers of p19A in the ceca, colons and colonic lumen of vancomycin-pretreated plus p19A-infected WT (n = 9) and *Sigirr -/-* mice (n = 12) were determined at day 5 post-infection. Data are representative of two independent experiments. (**c**) Bioluminescence signals from the large intestines (before and after washing with PBS) of vancomycin-pretreated, p19A-lux-infected WT and *Sigirr -/-* mice were imaged by an *in vivo* imaging system at day 5 post-infection. The color bar shown on the right indicates the strongest bioluminescence signal in red and the weakest bioluminescence signal in blue. The bioluminescence signal corresponds to logarithmic units of light measurement (photons/s/cm^2^/sr). Data are representative of two independent experiments. (**d**) Vancomycin-pretreated WT and *Sigirr -/-* mice were infected with p19A-GFP (expressing GFP on p19A chromosome) for 5 days, and the colonization of p19A-GFP on the cecal mucosal surface was visualized by immunostaining with antibodies against actin (red) and GFP (green). The nuclei were counterstained with DAPI (blue). Scale bar 20μm (D), and scale bar 100μm for (E) Data are representative of three independent experiments. (**e**) Representative H&E staining pictures of ceca of WT and *Sigirr -/-* mice (pretreated with vancomycin) at day 0 (n = 6 for both groups of mice) and day 5 (n = 9 for WT, n = 15 for *Sigirr -/-*) post p19A infection. Scale bar 20μm (D), and scale bar 100μm for (E) (**f**) Histopathological scores from cecal tissues of WT and *Sigirr -/-* mice used in (*E*). Data are representative of three independent experiments. *p < .05, **p < .01, ***p < .001.

### Sigirr -/- *mice show increased susceptibility to p19A colonization of the GI tract.*

Our group has previously shown that *Sigirr*-/- mice display heightened susceptibility to several intestinal pathogens^25,26^. We tested whether these mice would also be susceptible to p19A infection. WT and *Sigirr*-/- mice were pretreated with vancomycin for 6 hours and then infected with p19A. At 5 dpi, the numbers of p19A recovered from the ceca and colonic lumen of *Sigirr*-/- mice were almost 10 fold higher than those of WT mice, although similar levels of p19A were seen in the colons of both groups of mice (Figure 1b). We then examined the localization of p19A within the GI tract. Vancomycin-pretreated WT and *Sigirr*-/- mice were gavaged with a p19A derived luciferase-expressing strain (p19A-lux) for 5 days, followed by imaging of bioluminescence signals in excised intestinal tissues. Interestingly, strong luminescence signals were detected in the intact GI tracts (following euthanization) of both WT and *Sigirr*-/- mice (Figure 1c), but upon opening and vigorously washing away the luminal content from the intestinal tissues, the luminescence signals were largely lost from the ceca of WT mice, but remained strong in the ceca of *Sigirr*-/- mice. This suggests that the majority of p19A in WT mice are luminal, whereas p19A in *Sigirr*-/- mice may be adherent to the cecal mucosal surface. To confirm this, we infected vancomycin-pretreated WT, and *Sigirr*-/- mice with a p19A derived GFP-expressing strain (p19A-GFP), and collected cecal tissues for immunostaining. Antibodies against GFP, along with actin were used to visualize p19A-GFP bacteria and the intestinal epithelial surface, respectively. As shown in Figure 1d, GFP expressing bacteria were restricted to the cecal lumen of WT mice, whereas GFP positive bacteria were in close proximity to, or adherent to the cecal mucosal surface (indicated by arrows) of *Sigirr ^−/-^* mice.

We next examined if p19A colonization induced cecal inflammation in mice. While vancomycin-pretreated WT, and *Sigirr*-/- mice showed no inflammation in their ceca at baseline (Figure 1e), at day 5 post p19A infection, higher levels of inflammatory cell infiltration, increased submucosal edema and epithelial damage were seen in the ceca of *Sigirr*-/- mice as compared to WT mice (Figure 1e,f). Consistent with these microscopic changes, the mRNA levels of pro-inflammatory cytokines (*Il-6, Tnf-α*) in the cecal tissues of *Sigirr*-/- mice were double that of WT mice at 5 dpi (Figure S1). These data indicate that *Sigirr*-/- mice are susceptible to p19A colonization of their intestinal mucosal surface, with p19A adhering to their intestinal epithelial cells and inducing modest inflammation in their ceca.

### *p19A infection worsens subsequent DSS-induced colitis in vancomycin treated* Sigirr -/- *mice*

**Figure 2.**
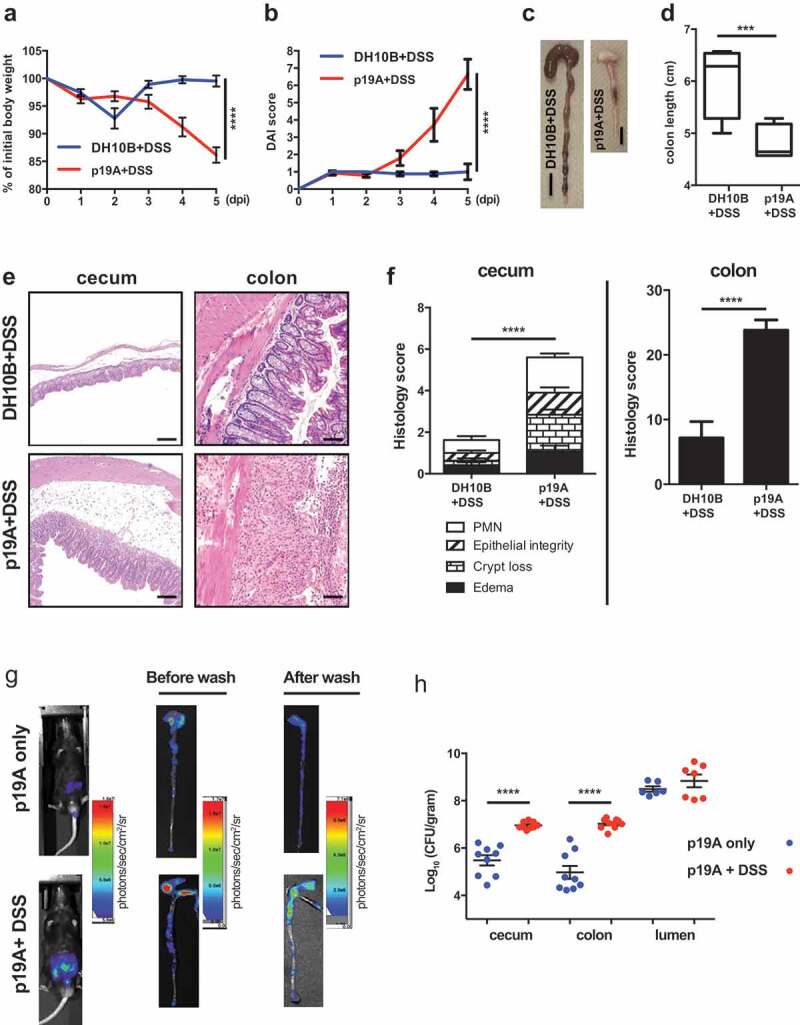
Acute infection with p19A worsens subsequent DSS-induced colitis in *Sigirr -/-* mice

Since p19A colonized the intestinal mucosal surface of *Sigirr*-/-mice, causing modest pathology, we asked whether infection with p19A would have an effect on subsequent intestinal inflammation induced by other noxious stimuli, such as dextran sodium sulfate (DSS). Notably, the DSS-induced colitis model has been widely used as a mouse model of human UC ^28^, mimicking many of the pathologic features seen in UC patient tissues. After testing a range of doses, we found that 2.5% DSS consistently caused a very mild colitis in *Sigirr*-/-mice in our animal facility. Vancomycin-pretreated *Sigirr*-/- mice were subsequently gavaged with the control *E. coli* DH10B (a derivative of commensal strain K-12) or p19A. One day after infection, mice were exposed to 2.5% DSS in their drinking water for another 4 days. Over the following days, body weights were recorded. As shown in Figure 2a, p19A+DSS treated mice lost significantly more body weight than DH10B+DSS treated mice, starting at 4 dpi. Consistent with this, the disease activity index (DAI) score (based on body weight loss, stool consistency and blood in the stool) of p19A+DSS treated mice was significantly higher than that of the DH10B+DSS treated groups at day 4 and 5 dpi (Figure 2b). When mice were euthanized at 5 dpi, a striking difference was noted in the large intestines of these mice. The cecal and colonic tissues of the DSS+ p19A treated mice were overtly shrunken and inflamed, and their colons were significantly shorter than those of mice given DH10B+DSS (Figure 2c,d).

(**a – b**) Vancomycin-pretreated *Sigirr -/-* mice were gavaged with p19A (n = 8) or *E. coli* DH10B (n = 14) for 1 day, then exposed to 2.5% DSS in drinking water for another 4 days, and their body weight (**a**) and disease activity index (DAI) (**b**) was recorded daily. Data are representative of six independent experiments. (**c**) Representative macroscopic images of their large intestines at day 5 post-infection showing shrunken cecum and shortened colon in p19A+DSS treated *Sigirr -/-* mouse as compared to *E. coli* DH10B+DSS treated mouse. Black scale bar, 1 cm. (**d**) A box and whisker plot showing colon length of *E. coli* DH10B+DSS or p19A+DSS treated *Sigirr -/-* mice. (**e**) Representative H&E staining images of ceca and colons of DSS-treated *Sigirr -/-* mice, previously infected with p19A or *E. coli* DH10B. Scale bar, 100 μm for (E) . (**f**) Histopathological scores from cecal and colonic tissues of mice used in (*D*). (**g**) Bioluminescence signals from the whole body and intestinal tissues (before and after wash with PBS) of mice used in (*D*) were imaged by *in vivo* imaging. (**h**) The numbers of p19A in ceca, colons and colonic lumen of WT and *Sigirr -/-* mice were determined at day 5 post-infection. These mice were pretreated with vancomycin for 6 h, and left unexposed (n = 6–9) or exposed to DSS treatment (n = 7–10) at day 1 post p19A infection. Data are representative of two independent experiments. ***p < .001, ****p < .0001.

Colonic shortening typically reflects severe mucosal inflammation. Thus, while H&E staining of cecal and colonic tissues of the DH10B+DSS treated mice revealed only mild inflammatory cell infiltration and epithelial cell damage, this was in stark contrast to the widespread inflammatory cell infiltration, severe edema, loss of crypt structures, and broad areas of deep ulceration seen in the p19A+DSS treated mice (Figure 2e). Correspondingly, the cecal and colonic pathology in the p19A+ DSS mice was significantly greater than that seen in the DH10B + DSS mice (figure 2f). We also examined levels of fecal lipocalin 2 (Lcn-2), since it functions as a sensitive, noninvasive marker for intestinal inflammation ^29^. Lcn-2 levels in the p19A+ DSS mice were almost two times those of the DH10B + DSS mice (Figure S2). We then examined if DSS-induced colitis affected the growth of p19A within the gut. Vancomycin-pretreated *Sigirr*-/- mice were infected with p19A-lux for 1 day, and exposed to DSS treatment for 4 days. *In vivo* imaging analysis revealed stronger bioluminescence signals emanating from DSS treated mice as compared to non-DSS treated mice (Figure 2g), and upon removal of the tissues, and repeated washing, stronger luminescence signals were detected in the ceca and colons of the DSS treated mice. Confirming this observation, when ceca and colons of these mice were collected for plating, the numbers of p19A bacteria recovered from the p19A+DSS treated mice was 2–4 log higher than that recovered from mice receiving p19A, but no DSS (Figure 2h). Notably, despite the inflammation, p19A were still found to adhere to areas of intact intestinal mucosa, an observation confirmed through electron microscopy (Figure S3). Collectively, these results demonstrate that prior infection with p19A aggravates DSS-induced colitis in vancomycin-pretreated *Sigirr*-/-mice, and this colitic response further promotes p19A growth in the gut.

### *UC-*E. coli *isolate p7 also aggravates subsequent DSS-induced colitis in* Sigirr -/- *mice.*

To test whether p19A’s ability to aggravate DSS-induced colitis was shared by other UC-associated pathobionts, we tested if prior infection with the UC isolate p7 intensified colitic responses induced by DSS. Notably, the pathobionts p7 and p19A both belong to the B2 phylogenetic group, but they display different sero-type profiles ^20^. Vancomycin pretreated *Sigirr*-/- mice were infected with *E. coli* DH10B or p7 for 1 day, followed by exposure to DSS as above. As shown in Figure S4, p7+ DSS treated mice showed significantly worsened clinical symptoms as compared to DH10B+DSS treated mice, including greater body weight loss, higher DAI, and exaggerated tissue pathology. Thus, *E. coli* isolates from different UC patients are conserved in their ability to worsen DSS-induced colitis in *Sigirr*-/- mice.

### p19A’s ability to worsen DSS-induced colitis is largely dependent on α-hemolysin

**Figure 3.**
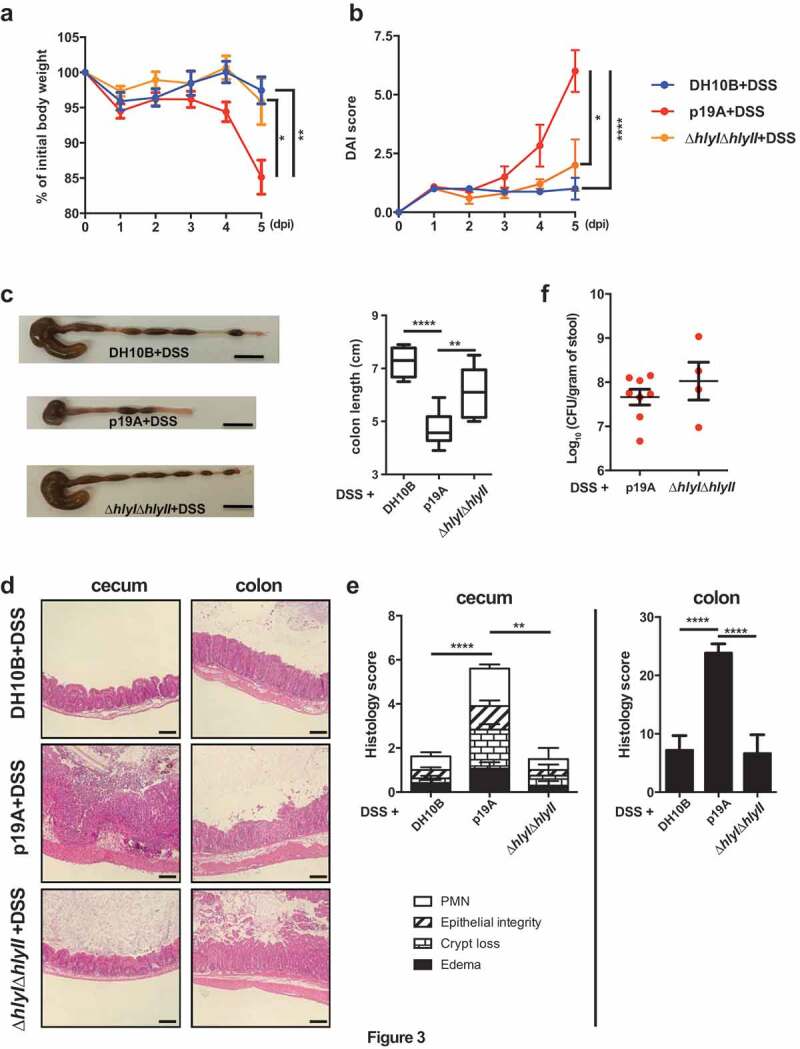
p19A’s ability to worsen DSS-induced colitis depends on two hemolysin genes

Our previous studies showed that much of the damage caused by p19A and other UC pathobionts to cells in culture was attributable to the production of α-hemolysin ^23^. To test whether the pro-colitic actions of p19A showed a similar basis, *Sigirr*-/- mice were pretreated with vancomycin and then gavaged with either wildtype p19A, or the ∆*hly*I∆*hlyII* mutant strain (a derivative of p19A that lacks hemolysin genes *hlyI* and *hlyII*). The ∆*hly*I∆*hlyII* mutant has been previously shown to suffer a significant loss of hemolytic activity^23^. As outlined earlier, at 1 dpi, the infected mice were given 2.5% DSS in their drinking water. Mice infected with the ∆*hly*I∆*hlyII* mutant showed significantly less body weight loss at 4 and 5 dpi, and a lower DAI score at 5 dpi (Figure 3a,b). Following euthanization, the mice infected with the ∆*hly*I∆*hlyII* strain showed less macroscopic intestinal damage (larger ceca and longer colons), and suffered significantly less histological damage than mice receiving wildtype p19A (Figure 3c-e). While reduced pathology and inflammatory response was seen in ∆*hly*I∆*hlyII* infected mice, the shedding of the ∆*hly*I∆*hlyII* mutant and wildtype p19A in the stool was comparable (Figure 3f). Thus, the *E. coli* pathobiont p19A worsens the course of DSS colitis through the production of α-hemolysin, a virulence factor previously linked to this microbe’s ability to trigger inflammatory responses *in vivo* and damage intestinal epithelial barrier function *in vitro* ^23, 30, 31^.

(**a and b**) Vancomycin-pretreated *Sigirr -/-* mice were infected with *E. coli* DH10B (n = 8), p19A (n = 8) or ∆*hly*I∆*hlyII* (a p19A derived strain lacking *hlyI* and *hlyII*) (n = 5) for 1 day, then exposed to 2.5% DSS in drinking water for another 4 days, and their body weight (**a**) and disease activity index (**b**) were recorded daily. Data are representative of three independent experiments. (**c**) Representative macroscopic images of the large intestines of mice at day 5 post-infection are shown in the left panel, and a box and whisker plot showing the colon length of these mice is displayed in the right panel. Black scale bar, 1 cm. (**d**) Representative H&E stained images of ceca and colons of DSS-treated *Sigirr -/-* mice previously infected with *E. coli* DH10B, p19A or ∆*hly*I∆*hlyII*. Scale bar, 100 μm. (**e**) Histopathological scores from cecal and colonic tissues of mice used in (*D*). (**f**) The number of wild type p19A or ∆*hly*I∆*hlyII* present in the stool was determined at day 5 post-infection. Data are representative of two independent experiments. *p < .05, **p < .01, ***p < .001, ****p < .0001.

### p19A’s potentiating effects on colitis depend on the adhesin FimH

**Figure 4.**
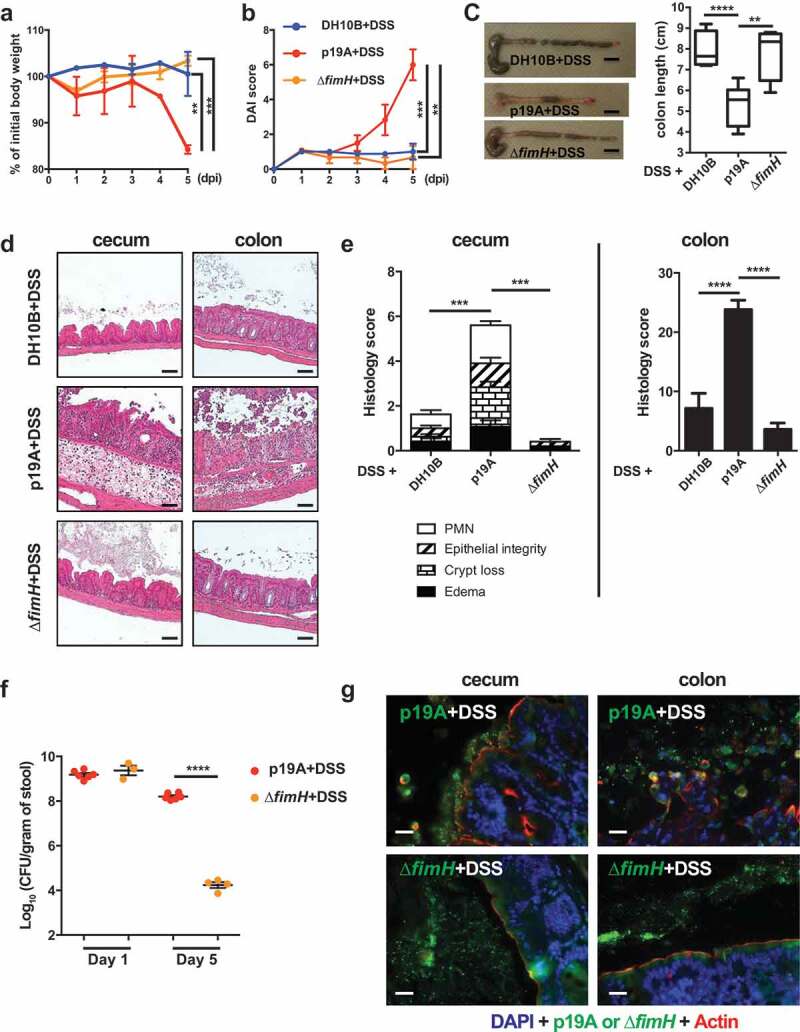
p19A’s potentiating effects on colitis depend on the adhesin FimH

The ability of p19A to adhere to the intestinal mucosal surface of *Sigirr*-/- mice suggests that p19A may express certain adherence factor(s) mediating its interaction with host cells. Indeed, the genome of p19A (unpublished data) consists of many genes encoding adherence factors, including pili and fimbriae ^32^. Of note, p19A contains *fimH*, a gene encoding a mannose-binding lectin named FimH. FimH is expressed by many other *E. coli* strains, including AIEC, and uropathogenic *E. coli* (UPEC), mediating their adherence to epithelial cells ^33,34,35^. Comparing amino acid sequences of FimH from different *E. coli* strains, we found that p19A FimH was phylogenetically most similar to *E. coli* K-12 FimH, containing two mutations (V51A and R190H) (Figure S5). To test whether FimH was a pathogenic factor of p19A, we generated an isogenic strain of p19A lacking *fimH* (∆*fimH*), and infected *Sigirr*-/- mice as outlined earlier. As shown in Figure 4a-e, compared to mice receiving wildtype p19A+DSS, ∆*fimH*+DSS treated mice showed significantly improved clinical symptoms, losing less body weight, displaying lower DAI scores, and suffering dramatically less macroscopic and histologic damage in their ceca and colons. Remarkably, the colitis developed in ∆*fimH*+DSS treated mice was similar to that seen in the control DH10B+DSS treated mice. To better understand how the mutation in *fimH* attenuates p19A pathogenicity, we compared p19A and ∆*fimH* burdens in infected mice. Although ∆*fimH* and wildtype p19A showed comparable levels of shedding in the stool at 1 dpi, by 5 dpi (4 days post-DSS), the levels of ∆*fimH* were dramatically reduced, to 4 logs lower (10^4^ CFU/gram) than those of wildtype p19A (10^8^ CFU/gram) (Figure 4f). Immunostaining of infected cecal and colonic tissues showed that wildtype p19A was present in both the lumen and on the intestinal epithelial surface, whereas ∆*fimH* was rarely detected, and only in the intestinal lumen (Figure 4g). These results suggest that FimH is not only critical for p19A to remain within the inflamed gut and be shed at high levels in the stool, but the mucosal adherence/lasting presence of p19A within the inflamed gut is also essential for p19A to aggravate DSS colitis.

(**a**) Vancomycin-pretreated *Sigirr -/-* mice were infected with *E. coli* DH10B (n = 5), p19A (n = 6) or ∆*fimH* (a p19A derivative strain lacking *fimH*) (n = 4) for 1 day, then exposed to 2.5% DSS in drinking water for another 4 days, and their body weight was recorded daily. (**b**) Disease activity index of *Sigirr -/-* mice as described in (*A*). Data are representative of three independent experiments. (**c**) Representative macroscopic pictures of the large intestines of *Sigirr -/-*mice as described above at day 5 post-infection (left panel), and a box and whisker plot showing the colon lengths of these mice (right panel). Black scale bar, 1 cm. (**d**) Representative H&E staining images of the ceca and colons of DSS-treated *Sigirr -/-* mice previously infected with *E. coli* DH10B, p19A or ∆*fimH*. Scale bar, 100 μm. (**e**) Histopathological scores of the ceca and colons from mice used in (*D*). (**f**) The number of p19A (n = 6) or Δ*fimH* (n = 3–4) bacteria in the stool was determined at day 1 and 5 post-infection. (**g**) Localization of p19A and ∆*fimH* in ceca of mice used in (*A*). p19A and ∆*fimH* bacteria were visualized by immunofluorescent staining with anti-actin (red) and anti-LPS (green). The nuclei were stained with DAPI (Blue). Scale bar, 20 μm. Data are representative of two independent experiments. **p < .01, ***p < .001, ****p < .0001.

### FimH antagonists prevent p19A from aggravating DSS-induced colitis

**Figure 5.**
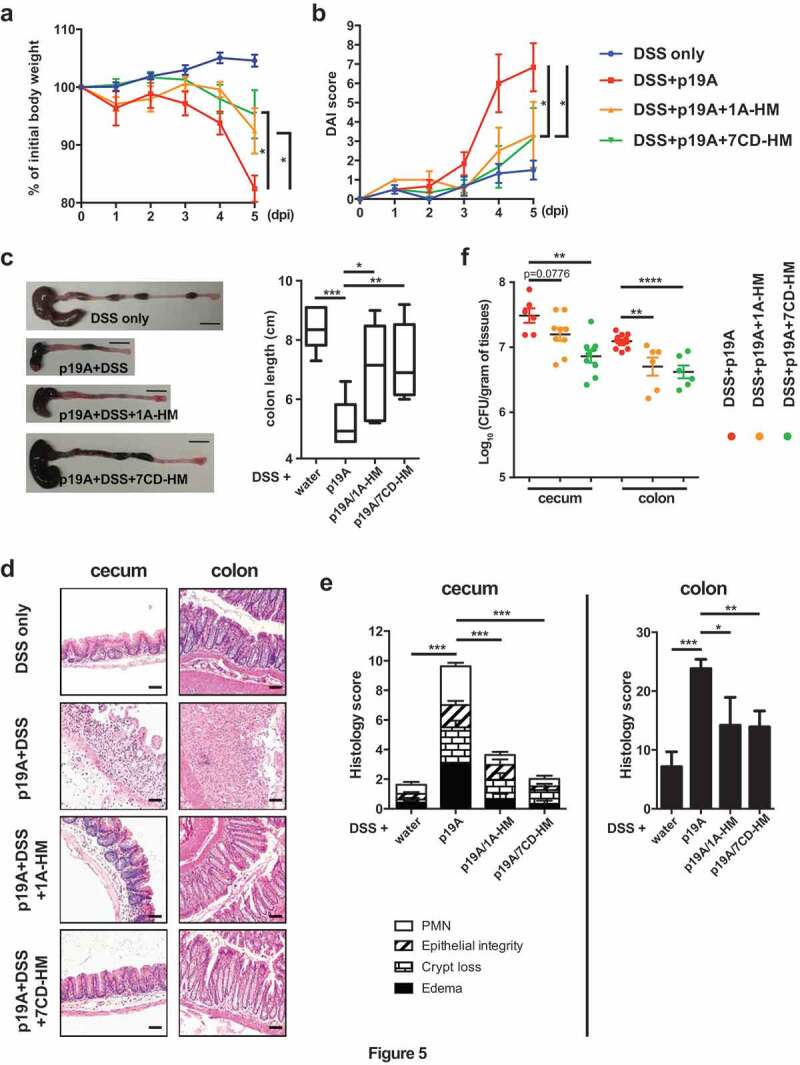
FimH antagonists prevent p19A from aggravating DSS-induced colitis

FimH contains a carbohydrate recognition domain (CRD) that interacts with mannosylated proteins expressed on the surface of epithelial cells^36^. FimH antagonists mimicking the natural ligands of FimH have been developed to saturate this CRD, thus inhibiting the binding of FimH^37,38^. We were therefore interested in determining whether these antagonists would diminish the ability of p19A to potentiate colitis. Vancomycin treated *Sigirr*-/- mice were infected with p19A or DH10B, followed by oral administration of PBS (vehicle control) or different FimH antagonists (1A-HM and 7 CD-HM, 10 mg/kg per mouse)^37^ at 2 h post-infection, as well as at days 1, 2 and 3 post-infection. 1A-HM ^39^and 7 CD-HM ^40^are structural analogues of heptyl-α-D-mannoside (HM), a nanomolar FimH antagonist. 1A-HM is an optimized monovalent HM derivative bearing an isopropylamide group while 7 CD-HM is an heptavalent glycoconjugate where the HM ligands are grafted onto a β-cyclodextrin core. At 1 dpi, DSS was given to these mice as outlined earlier, and they were monitored for another 4 days. As shown in Figure 5a-e, mice receiving either antagonist showed significantly less weight loss, lower DAI scores, as well as less macroscopic and microscopic damage, although 7 CD-HM appeared to be more effective than 1A-HM. To determine if these FimH antagonists affected the interaction of p19A with intestinal epithelial cells, cecal and colonic tissues were opened and washed extensively, followed by homogenization and plating. The numbers of p19A recovered from cecal and colonic tissues of antagonist-treated mice were significantly lower than those recovered from vehicle treated mice (Figure 5f), suggesting that p19A were prevented from adhering to the intestinal mucosa in the presence of antagonists. Thus, FimH antagonists, by inhibiting the adherence of p19A to the intestinal mucosa, reduced p19A’s ability to aggravate DSS-induced colitis.

(**a**) Vancomycin-pretreated *Sigirr -/-* mice were uninfected, or infected with p19A for 1 day, then exposed to 2.5% DSS in drinking water for another 4 days. Also, the FimH antagonists 1A-HM and 7 CD-HM were orally gavaged into mice at 2 h post-infection, as well as on day 1, 2, and 3 post-infection. The body weights of these mice were recorded daily (n = 6, 5, 8 and 8, for mice receiving DSS only, DSS+p19A, DSS+p19A+1A-HM, and DSS+p19A+7 CD-HM, respectively). (**b**) Disease activity index of *Sigirr -/-* mice as described in (*A*). (**c**) Left panel, representative macroscopic pictures of the large intestines of *Sigirr −/-*mice as described above; Right panel, a box and whisker plot showing the colon lengths of these mice. Black scale bar, 1 cm. (**d**) Representative H&E staining images of the ceca and colons of DSS-treated *Sigirr -/-* mice that were previously infected with p19A and orally gavaged with the FimH antagonists 1A-HM or 7 CD-HM. Scale bar, 100 μm. (**e**) Histopathological scores of ceca and colons from mice used in (*D*). (**f**) The numbers of p19A bacteria in ceca and colons of mice receiving DSS+p19A (n = 6–12), DSS+p19A+1A-HM (n = 6–9), or DSS+p19A+7 CD-HM (n = 6–9), were determined at day 5 post-infection. Data are representative of three independent experiments. *p < .05, **p < .01, ***p < .001, ****p < .0001.

### Discussion

The etiology of IBD is complex, and appears to involve the interaction of genetic, environmental and immunologic factors. Also, luminal bacteria or their products play a critical role in the instigation and exacerbation of the chronic intestinal inflammation seen in IBD ^10,11,41,42^. For instance, AIEC is associated with CD, whereas a variety of recently isolated B2 phylogenetic group *E. coli* strains are associated with UC^20,31^. Many *in vitro* and *in vivo* models have been developed to define how AIEC pathogenicity factors and the host response to this microbe act in concert to potentially contribute to the etiology of CD. In contrast, the potential contribution of B2 phylogroup *E. coli* strains to UC has received less attention, with their pathogenesis primarily studied in cell culture^23^. Robust animal models that utilize these UC associated *E. coli* strains in a relevant fashion are clearly needed. Here, we found that the clinically isolated UC pathobiont p19A was able to colonize the intestines of genetically susceptible mice (*Sigirr*-/-) following antibiotic treatment. While p19A caused only modest intestinal inflammation on its own, it was found to adhere to the intestinal mucosal surface and worsen subsequent DSS-induced colitis. Using this robust animal model, we also characterized key pathogenicity factors of p19A involved in colitis development.

Our results indicate an increased susceptibility of *Sigirr* -/- mice (compared to C57BL/6 mice) to p19A infection following antibiotic treatment. This may not be surprising, considering that this mouse strain is also more susceptible to many other enteric pathogens, including *C. rodentium, S*. Typhimurium and *C. jejuni*^25,26^. Upon infection with these pathogens, *Sigirr* -/- mice display exaggerated inflammatory and/or antimicrobial responses that promote their expansion and colonization in the gut. *Sigirr* -/- mice infected with p19A also exhibited slightly higher inflammatory responses than WT mice, as well as modest gut inflammation. We reason that p19A causes only modest inflammation on its own because it does not express the typical pathogenicity factors used by intestinal bacterial pathogens to induce strong inflammatory responses, such as a type III secretion system and the capsular polysaccharides expressed by many Gram-negative bacterial pathogens^43,44^. From an evolutionary perspective, lacking the expression of these factors may benefit p19A, allowing this microbe to survive and thrive within the GI tract without excessively agitating its host.

Vancomycin pretreatment enabled p19A to heavily colonize the intestines of both C57BL/6 mice and *Sigirr* -/- mice, suggesting that the microbial dysbiosis caused by antibiotic treatment created an environment that favored the expansion of p19A in both mouse strains. Even so, immunostaining of intestinal tissues showed that p19A was able to adhere to the intestinal mucosa of *Sigirr* -/- mice (but not C57BL/6 mice). The basis for this difference is unclear, but could reflect the greater inflammatory response in p19A+vancomycin treated *Sigirr* -/- mice (as compared to C57BL/6 mice). Similar to the report by Winter et al. ^45^, the heightened inflammatory tone of *Sigirr* -/- mice may differentially induce p19A’s virulence factors, allowing it to colonize the surface of the inflamed gut. It is also possible there are key factors expressed on the inflamed intestinal mucosa of *Sigirr* -/- mice that permit p19A binding. For example, AIEC infection has been shown to up-regulate the expression of the carcinoembryonic antigen related cell adhesion molecule (CEACAM6) by intestinal epithelial cells, thus facilitating its adherence to the ileal mucosa of CD patients^18^. The fact that antibiotic pretreatment alone was sufficient to increase p19A colonization levels in the gut suggests a potential for p19A to worsen already existing UC, or exacerbate disease relapses in the clinical setting. Consistent with our findings in animal studies, p19A and other similar *E. coli* pathobionts are often seen in UC patients with inactive, as well as active disease^20,31^.

While p19A infection alone caused only modest intestinal inflammation in *Sigirr* -/- mice, it dramatically potentiated subsequent DSS-induced inflammation. Moreover, p19A numbers bloomed during DSS-induced intestinal inflammation, as evidenced by the recovery of significantly higher numbers of p19A from the ceca and colons of p19A+DSS treated mice as compared to mice not receiving DSS. These data suggest that p19A, or other UC pathobionts could dwell at low numbers within the gut, but upon the onset of intestinal inflammation triggered by other noxious stimuli, take advantage of the inflammation and expand to aggravate disease. Interestingly, the pro-colitic effect of p19A was also seen with another B2 phylogenetic group *E. coli* strain termed p7. Whether other UC-associated *E. coli* strains show similar pro-colitic effects awaits further investigation.

The ability of p19A to potentiate colitis depends on key pathogenic factors. One of these is α-hemolysin, a pore forming toxin that can cause cell lysis and induce tissue damage. Notably, α-hemolysin-expressing *E. coli* strains are often found in UC biopsies ^30,32^. We and others have shown that many *E. coli* strains (including p19A), through α-hemolysin production, disrupt tight junctions and cause focal lesions in cultured epithelial cells ^23,46^. Correspondingly, we found that the ∆*hlyI* ∆*hlyII* mutant of p19A lost its ability to potentiate DSS-induced colitis. Using a mouse model that does not involve DSS treatment, Bucker et al. ^30^ have also demonstrated that *E. coli* α-hemolysin impairs intestinal barrier function, although this is through the induction of focal lesions in the colonic epithelium. Thus, both mouse models highlight the potential for p19A’s α-hemolysin to cause tissue damage and/or intensify ongoing inflammation and disease.

Previous studies have found that the FimH adhesin aids other *E. coli* strains in attaching to epithelial cells and adapting to different environmental conditions ^47,48,49^. Building on these findings, our study identified FimH as an essential pathogenicity factor for p19A. The ∆*fimH* mutant (derived from wildtype p19A) failed to colonize the intestinal mucosal surface. Moreover, mice treated with ∆*fimH*+DSS showed almost no tissue pathology at 5 dpi. Similar to our findings, inactivation of *fimH* in various extraintestinal pathogenic *E. coli* strains (including UPEC and AIEC) decreases their colonization in the gut and/or reduces their ability to promote inflammation^34,50,51,52,53^. We also saw a remarkable decrease in the fecal shedding of the ∆*fimH* mutant from infected *Sigirr* -/- mice over time. One possible explanation is that the *∆fimH* mutant may be unable to attach to the intestinal mucosal surface, and is flushed out of the intestinal lumen by the flow of feces, water or mucus. Alternatively, since FimH is important for biofilm formation in many other bacteria^54,55^, the ∆*fimH* mutant may have been unable to form biofilms in the gut, and was thus unable to persistently colonize the inflamed intestine. Interestingly, FimH-mediated adhesion of UPEC to the urothelial surface causes the activation of the nuclear factor-κB (NF-κB) signaling pathway, leading to the secretion of pro-inflammatory cytokines ^56^. In future studies, it will be interesting to determine whether p19A also aggravates DSS-induced colitis through FimH-dependent activation of NF-κB signaling.

While there are only two point mutations separating the FimH of p19A from that of the commensal *E. coli* K-12, we speculate that these mutations are sufficient to mediate p19A’s ability to adhere to the intestinal mucosal surface of *Sigirr* -/- mice and worsen their course of colitis. Supporting this, point mutations in FimH also confer AIEC bacteria a higher ability to persist and induce intestinal inflammation in genetically susceptible transgenic mice ^34^. Therefore, as proposed previously^34^, analyzing single nucleotide polymorphisms of *fimH* could potentially predict the pathogenicity of *E. coli* strains isolated from CD and UC patient biopsies.

To further characterize the role of FimH in p19A pathogenicity, we treated *Sigirr* -/- mice with two FimH antagonists, and found that they significantly improved the clinical symptoms induced by p19A+DSS treatment. The colonization by p19A of the intestinal mucosa was significantly reduced in mice treated with these antagonists. It is possible that these antagonists are able to inhibit the interaction of FimH with mannosylated proteins on the intestinal mucosa, but have no or minimal effect on the ability of p19A to form biofilms *in vivo* (since the fimbriae structure is still present). In addition to suppressing the pathogenicity of p19A in our colitis model, FimH antagonists have been shown to alleviate AIEC induced colitis ^37^ and deplete UPEC from infected mice ^38^. Thus, FimH represents a very promising therapeutic target in the fight against intestinal infection/inflammation caused by a wide range of *E. coli* strains, including UC-associated *E. coli* strains. The FimH antagonists targeting one type of *E. coli* could prove broadly effective, as seen for 1A-HM and 7 CD-HM, which also attenuated the pathogenicity of AIEC LF82 in a different mouse model of colitis ^37^.

Taken together, we have developed a robust mouse model of colitis through the use of the UC-associated *E. coli* p19A. While it is conceivable that similar pathobionts may initiate UC in susceptible individuals, it appears more likely that they arise in the context of intestinal inflammation, exacerbating and promoting the chronicity of the disease, as well as potentially converging with other inflammatory stimuli to promote disease relapse. We further show the pro-colitic effects of p19A are mediated by at least two key factors – α-hemolysin and FimH. Selectively inhibiting FimH activity using antagonists prevented p19A from worsening colitic responses. These findings led us to propose a working model whereby p19A contributes to UC development in genetically susceptible hosts (Figure S6). While p19A is isolated from UC patients, LF82 is isolated from CD patients. They both belong to the B2 phylogenetic group and use FimH to potentiate colitis. However, in contrast to p19A, LF82 does not exhibit hemolytic activity or cause tight junction disruption^23^. Future work will be needed to address other genetic similarities and differences between these two pathobionts. Nevertheless, our pre-clinical model should not only facilitate studying how bacterial-host interactions play a role in the pathogenesis of UC, but will also significantly advance the development of novel therapeutic strategies that target UC.

## Materials and methods

### Bacterial strains

UC-associated *E. coli* B2 strains p19A and p7, and the ∆*hly*I∆*hlyII* mutant (generated from p19A) lacking both hemolysin genes, have been described previously^20,23^. A p19A-derived ∆*fimH* mutant was generated by allelic exchange using the gene doctoring technique as previously described ^57^. Briefly, primers GDup*fimH* and GDdw*fimH* (primer sequences are summarized in Table 1) were used to amplify a kanamycin cassette from the plasmid pDOC-K. The GDup*fimH* primer contains 50 bp of homology to the DNA sequence upstream of *fimH* and 20 bp of sequence (K-FWD) from pDOC-K. The GDdw*fimH* primer contains 50 bp of homology to the sequence of the terminal region of *fimH* and 18 bp (*P*-REV) of sequence on pDOC-K. The amplified kanamycin cassette was cloned into the plasmid pDOC-C, which was subsequently transformed into p19A carrying the helper plasmid pACBSCE encoding the λ-Red recombinase. To induce recombination, transformed bacteria were grown in Luria-Bertani (LB) broth containing 0.5% L-arabinose. Recombinant bacteria were further grown on LB agar containg 50 μg/ml kanamycin and 5% sucrose to select for the ∆*fimH* mutant. Correct allelic replacement of the wild type *fimH* leaving only a 39 bp scar of the *fimH* gene was verified by PCR. The functional loss of FimH by the ∆*fimH* mutant was also confirmed with a yeast agglutination assay as described by others ^58^.

Since p19A is susceptible to many different antibiotics, it is difficult to selectively recover it from p19A-infected tissues. To circumvent this limitation, we inserted a chloramphenicol resistance marker on the chromosome of wildtype p19A. Briefly, a triple mating involving wildtype p19A, *E. coli* MFDλ*pir* containing pMAC5 (a chloramphenicol-marked Tn*7* delivery vector) ^59,60^, and *E. coli* MFDλ*pir* containing a helper plasmid pTNS2 ^61^ was performed on LB agar containing diaminopimelic acid (DAP). Conjugants were selected by plating 24 h conjugation mixture onto LB agar containing chloramphenicol but lacking DAP, and screened for the proper Tn7 transposition as before^61^. The p19A strain carrying the chloramphenicol resistance marker in the right position of the chromosome was named p19A-Chl^r^. Using the same method, we also generated a p19A-lux strain expressing the the *Photorhabdus luminescens lux* operon ^62^, and a p19A-GFP strain expressing GFP on the chromosome of p19A. The *lux* operon or *gfp* was expressed under the control of the promoter PLtetO^63,64^. Notably, p19A-Chl^r^, p19A-GFP, p19A-lux and the parental strain p19A showed similar potential to potentiate DSS colitis (data not shown). Note that all bacterial strains were grown from single colonies on LB plates, and cultured in 5 ml of LB broth without antibiotics, or with chloramphenicol (30 μg/ml), kanamycin (50 μg/ml), or DAP (0.3 mM) at 37°C overnight with shaking at 200 rpm. The next day, overnight cultures were subcultured at 1:250 dilution into 5 ml of LB at 37°C at 200 rpm until the OD_600_ values reached 0.8 ~ 0.85, followed by centrifugation of 1 ml of the cultures at 4000 rpm for 5 min. Culture pellets were resuspended in 1 ml of sterile PBS, and 100 μl of the suspension was used for oral infection of mice as detailed below.

### Mouse infection experiments

The C57BL/6 mice (originally from Charles River Laboratories) and the single immunoglobulin and toll-interleukin 1 receptor (TIR) domain (*Sigirr*) -/- mice used in these studies were bred and maintained under specific pathogen-free conditions at BC Children’s Hospital Research Institute. All mice were housed in a temperature-controlled (22 ± 2°C) animal facility with a 12-h light-dark cycle. Male mice at 6–10 weeks of age were orally gavaged with 0.1 ml of a 50 mg/ml vancomycin solution suspended in PBS (5 mg per mouse). Six hours later, each mouse was infected by oral gavage with approximately 7 × 10^7^ CFU of wildtype p19A, p19A-Chl^r^, p19A-GFP, p19A-lux, ∆*hly*I∆*hlyII*, ∆*fimH*, or *E. coli* DH10B. One day after infection, mice were exposed to 2.5% dextran sulfate sodium (DSS) in their drinking water. For the FimH antagonist experiment, 1A-HM and 7 CD-HM were orally administered in a volume of 0.1 ml of PBS at a dose of 10 mg/kg, at 2 h, day 1, 2, and 3 post-infection. Disease activity index (DAI) scores were recorded according to the following criteria (body weight, stool consistency and blood in the stool): Score 0 – no weight loss, hard stool and no blood detection; Score 1 – less than 10% body weight loss, hard stool and no blood in the stool; Score 2–10 − 15% body weight loss, loose stool and fecal occult blood; Score 3 – 15 – 20% body weight loss, loose stool with gross blood; Score 4 – > 20% body weight loss, diarrhea and rectal bleeding. A maximum score was 12. Mice were euthanized at day 4 or 5 post-infection, and tissues collected for further analysis.

### Ethics statement

All procedures involving the care and handling of the mice were performed according to protocol number A15-0206, approved by the University of British Columbia’s Animal Care Committee and in direct accordance with the Canadian Council of Animal Care (CCAC) guidelines. Mice were monitored daily for mortality and morbidity throughout their infection and euthanized if they showed signs of extreme distress or more than 20% body weight loss.

### Histology and pathological scoring

Tissues fixed in 10% formalin were paraffin embedded and cut for further histological analysis. The paraffin embedded tissue sections were stained with hematoxylin and eosin, photographed, and then used for pathological scoring. The scoring was done by two blinded observers. The scoring scheme was slightly different depending on the animal models and tissues used. For cecal tissues infected with p19A but not treated with DSS, the following scoring scheme was used: (1) submucosal edema (0-no change; 1-mild (<50% of the diameter of the entire intestinal wall); 2-moderate (50 ~ 80%); 3, severe (>80%)), (2) crypt hyperplasia (0-no change, 1: 1–50%, 2: 51–100%, 3: >100%), (3) epithelial integrity (0-no pathological changes detectable, 1-epithelial desquamation (few cells sloughed, surface rippled), 2-erosion of epithelial surface (epithelial surface rippled, damaged), 3-epithelial surface severely disrupted/damaged, large amounts of cell sloughing), (4) PMN cell infiltration (per 400× magnification field) (0- no change <5; 1–5 ~ 20; 2–20 ~ 50; 3- >50 cells/field). A maximum score under this scale was 12. For cecal tissues exposed to DSS treatment (regardless of infection with p19A), the scoring scheme included: submucosal edema, epithelial integrity and PMN cell infiltration, as described above; and crypt loss (0-none; 1-the basal 1/3 crypt loss; 2-the basal 2/3 portion damaged; 3-the entire crypt damaged but the surface epithelium remained intact; 4-the entire crypt and epithelium lost). A maximum score under this scale is 13. For colonic tissues recovered from DSS-induced colitis model, the entire colon was divided into 6 parts (2 from proximal, 2 from middle and 2 from distal colon), and then each part was assessed (0–7 for maximum) and summed up for individual mouse (total 0 to 42 for maximum). The scoring scheme included: crypt loss (0–4) as above, and inflammatory cell infiltration (0-rare; 1-inflammatory cells in lamina propria; 2-inflammatory cells in both lamina propria and submucosa; 3-transmural extension of infiltrate).

### Bacterial counts

To enumerate bacteria within large intestinal tissues and lumen, the ceca and colons were opened longitudinally, and the luminal contents were collected in a 2.0 ml microtube. Tissues were washed in PBS extensively, before being placed in the microtube. Tissue and luminal contents were homogenized in a MixerMill 301 bead miller (Retsch) for a total of 6 mins at 30 Hz at room temperature. Stool samples were similarly processed to count bacteria in the feces. Homogenized tissues, luminal contents or fecal samples were plated onto LB agar containing chloramphenicol (30 μg/ml) for p19A WT strains, or kanamycin (50 μg/ml) for ∆*hly*I∆*hlyII* and ∆*fimH* mutant strains. After culturing at 37°C overnight, bacterial counts were recorded.

### In vivo imaging

In vivo imaging was performed using the Ami-x platform (Spectral Instruments Imaging, AZ). Grayscale reference images taken under low illumination were collected and overlaid with images capturing the emission of photons from the *lux*-expressing p19A-lux using AMIView software. Live mice were anesthetized and images were taken. Thereafter, the mice were euthanized, followed by imaging of the intestinal tissues (before and after washing with PBS).

### Immunofluorescent staining of intestinal tissues

Following a previously described protocol^65^, paraffin sections (5 μm) were deparaffinized by heating at 55–65°C for 10 min, cleared with xylene, rehydrated through an ethanol gradient to water. Sections were then blocked in blocking buffer (2% donkey serum in PBS containing

0.1% Triton-X100 and 0.05% Tween 20). The primary antibodies used were anti-actin (1:100, clone I-19, Santa Cruz), anti-GFP (1:100, GeneTex), and anti-O6 LPS of p19A (1:80 *E. coli* O6 antiserum raised in rabbits, statens serum institute, Demark). Secondary antibodies used were donkey anti-goat Alexafluor-568 or donkey anti-rabbit Alexafluor-488 (1:2000, Life Technologies). Tissues were mounted using ProLong Gold Antifade reagent (Molecular Probes/Invitrogen), and nuclei were counterstained with 4ʹ, 6ʹ-diamidino-2-phenylindole (DAPI). Sections were viewed at 350, 488, and 594 nm on a Zeiss AxioImager microscope. Images were obtained using a ZeissAxio Imager microscope equipped with an AxioCam HRm camera operating through AxioVision software (Version 4.4).

#### Electron microscopy (EM) fixation and imaging

*Sigirr*-/- mice were pre-infected with p19A for 1 day, and then exposed to 2.5% DSS in their drinking water. After 2 days of DSS exposure, cecal and colonic tissues of p19A infected mice were removed and processed. EM studies of tissue samples and overnight p19A cultures (37°C in LB) were performed as previously described ^66^.

### Real time qPCR analysis

Total RNA from cecal and colonic tissues was extracted using the Qiagen RNeasy kit following the manufacturer’s instructions, and quantified using a NanoDrop Spectrophotometer (ND1000). 1 μg of RNA was reverse-transcribed using a Qiagen OmniscriptRT kit (Qiagen), followed by dilution of the cDNA at 1:5 in RNase/DNasefree H_2_O. The qPCR was carried out using a Bio-Rad CFX connect Real-time PCR detection system, with the specificity for each of the PCR reactions confirmed by melting point analysis. Quantitation was performed using CFX Maestro^TM^ software (Bio-Rad). The expression of genes was normalized to that of *Rplp0*. Primer sequences and reaction conditions for all genes analyzed are summarized in Table 1.

**Table 1.** Primers used for qPCR and construction of mutants

Gene	Forward 5’-3’	Reverse 5’-3’
*Rplp0*	AGATTCGGGATATGCTGTTGGC	TCGGGTCCTAGACCAGTGTTC
*Il-6*	GAGGATACCACTCCCAACAGACC	AAGTGCATCATCGTTGTTCAT
*Tnf-α*	CATCTTCTCAAAATTCGAGTGACAA	TGGGAGTAGACAAGGTACAACCC
GDup*fimH*	5’-GATACAGGATTCCATTCAGGCAGTGATTAGCATCACCTATACCTACAGCTGAACCCGAAGAG GACCGGTCAATTGGCTGGAG-3’	
GDdw*fimH*	5’-GATACAGTCGACCTGTGATTTCTTTATTGATAAACAAAAGTCACGCCAATAATCGATTGCAC AATATCCTCCTTAGTTCC-3’	

### Lipocalin-2 levels in the stool

One or two stool pellets (average weight 40 ± 10 mg) were freshly collected and resuspended at the concentration of 50 mg/ml in cold PBS containing proteinase inhibitor (Roche). The samples were homogenized with metal beads in a MixerMill 301 bead miller (Retsch) for 6 minutes at 30 Hz. Homogenized samples were centrifuged at 12,000 rpm, 4°C for 15 minutes. Supernatants were collected and stored at −80°C until use. To measure stool Lcn-2 levels, mouse lipocalin 2 DuoSet ELISA kit (R&D Systems) were used according to the manufacturer’s instructions.

### Statistical analysis

The results were analyzed using GraphPad Prism 8. Statistical significance was determined using a two-tailed Student’s t test. Most results presented are expressed as the mean value ± standard error of the mean (SEM) unless otherwise stated. * *p* < .05, ** *p* < .01, *** *p* < .001, **** *p* < .0001.

## Supplementary Material

Supplemental Material

## Data Availability

All data generated and reagents are available from the corresponding author on reasonable request.
