# Supplementary material for: Ulcerative Colitis-associated E. coli pathobionts potentiate colitis in susceptible hosts
Source: Gut Microbes. 2020 Dec 1;12(1):1847976. doi: 10.1080/19490976.2020.1847976 (PMC7781664; doi:10.1080/19490976.2020.1847976)
Supplement: Supplemental Material [file KGMI_A_1847976_SM9787.zip › Supplementary information/Supplementary Figures LEGENDS.docx]

**Supplementary Figures**

**Figure S1. *Sigirr -/-* mice show increased susceptibility to p19A colonization of the GI tract.**

The relative expression of pro-inflammatory cytokines (*Il-6* and *Tnf-α*) in cecal tissues of C57BL/6 mice and *Sigirr -/-*mice (Day 0 and Day 5 post-infection) was assessed by real-time PCR analysis. Data were normalized against the expression of the housekeeping gene *Rplp0* (n=6 for WT and *Sigirr -/-* at day 0; n=5 for WT at day 5; n=8-9 for *Sigirr -/-* at day 5). Data are representative of two independent experiments. *p<0.05, **p<0.01.

**Figure S2. *Sigirr -/-* mice treated with p19A plus DSS show increased fecal Lcn-2 levels.**

Vancomycin-pretreated *Sigirr -/-* mice were gavaged with p19A or *E. coli* DH10B (n=8 for both groups) for 1 day, then exposed to 2.5% DSS in drinking water for another 4 days, and fecal Lcn-2 levels at 5 dpi were measured by ELISA. **p<0.01.

**Figure S3.** **p19A adheres to the intestinal mucosal surface of *Sigirr -/-* mice.**

(**A**) Electron micrograph of p19A recovered from overnight cultures (grown in LB). (**B**) Electron micrograph of p19A recovered from the colonic lumen of a vancomycin-pretreatd *Sigirr -/-* mouse infected with p19A. Electron micrgraph of p19A recovered from the cecum (**C**) and colon (**D**) of *Sigirr -/-* mice that were treated with vancomycin 6 h before infection, infected with p19A for 1 day and exposed to 2.5% DSS treatment for another 2 days. Pictures in the right panels of (*C*) and (*D*) correspond to the boxed areas in the left panels of (*C*) and (*D*), respectively, which show that p19A bacteria are in close proximity to the microvilli of intestinal epithelial cells.

**Figure S4. The UC-*E.coli* isolate p7 also aggravates DSS-induced colitis in *Sigirr -/-* mice.**

(*A-E*) Vancomycin-pretreated *Sigirr -/-* mice were infected with DH10B (n=7) or p7 (n=7-8) (another *E. coli* strain isolated from a UC patient) for 1 day, then exposed to 2.5% DSS in drinking water for another 3 days, and their body weights (**A**) and disease activity index (**B**) were recorded daily until 4 dpi. (**C**) Left panel, representative macroscopic images of the large intestines of mice at 4 dpi; Right panel, a box and whisker plot showing the colon lengths of these mice at 4 dpi. Black scale bar, 1 cm. (**D**) Representative H&E staining pictures of ceca and colons of mice at 4 dpi. Scale bar, 100 m. (**E**) Histopathological scores of ceca and colons at 4 dpi. Data are representative of two independent experiments. *p<0.05, **p<0.01, ***p<0.001.

**Figure S5.** **Sequence** **analysis of FimH from different *E. coli* strains.** (**A**) Alignment of FimH sequences from AIEC strains LF82 and NRG857c, UPEC CFT073, *E. coli* K-12 and p19A. (**B**) Phylogenetic analysis of FimH sequences from the above bacteria by Vector NTI software (Thermo Fisher).

**Figure S6.** **A proposed working model illustrating how p19A contributes to UC development.**

(Left panel) In WT mice, orally gavaged p19A remains in the intestinal lumen, and is unable to attach to intestinal epithelial cells. These mice maintain intestinal homeostasis and do not develop overt intestinal pathology or inflammation. (Middle panel) In contrast, in genetically susceptible mice (*i.e.* *Sigirr*-/- mice), p19A attaches to the surface of intestinal epithelial cells and aggravates DSS-induced colitis, and this effect is mediated by at least two key factors - α-hemolysin and FimH. (Right panel) Selectively inhibiting FimH activity using antagonists prevents p19A from worsening DSS-induced colitic responses. This figure is created with Biorender.com.
