# Supplementary figures and images for: Ulcerative Colitis-associated E. coli pathobionts potentiate colitis in susceptible hosts
Source: Gut Microbes. 2020 Dec 1;12(1):1847976. doi: 10.1080/19490976.2020.1847976 (PMC7781664; doi:10.1080/19490976.2020.1847976)

**A**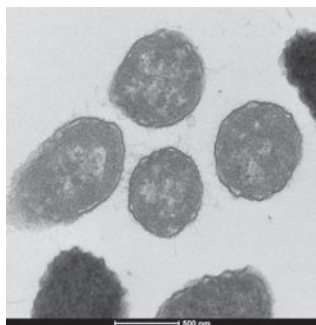**B**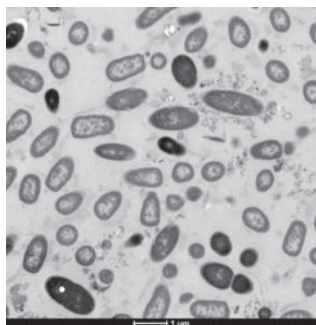**C**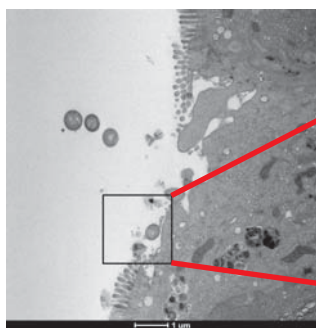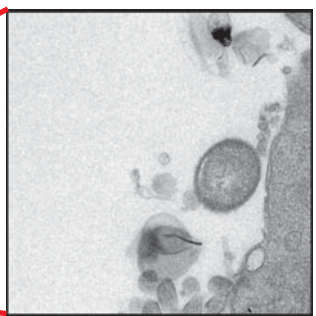**D**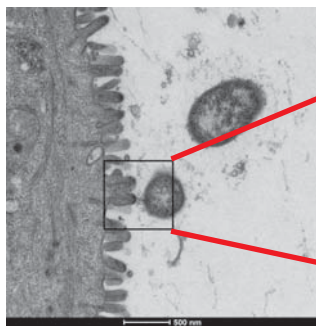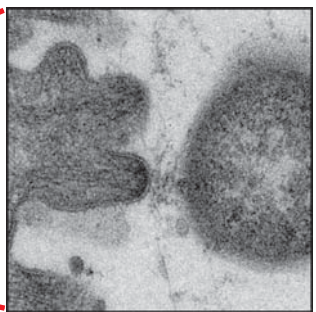

**Figure S3**

Supplement: Supplemental Material [file KGMI_A_1847976_SM9787.zip › Supplementary information/Figure S3.pdf]

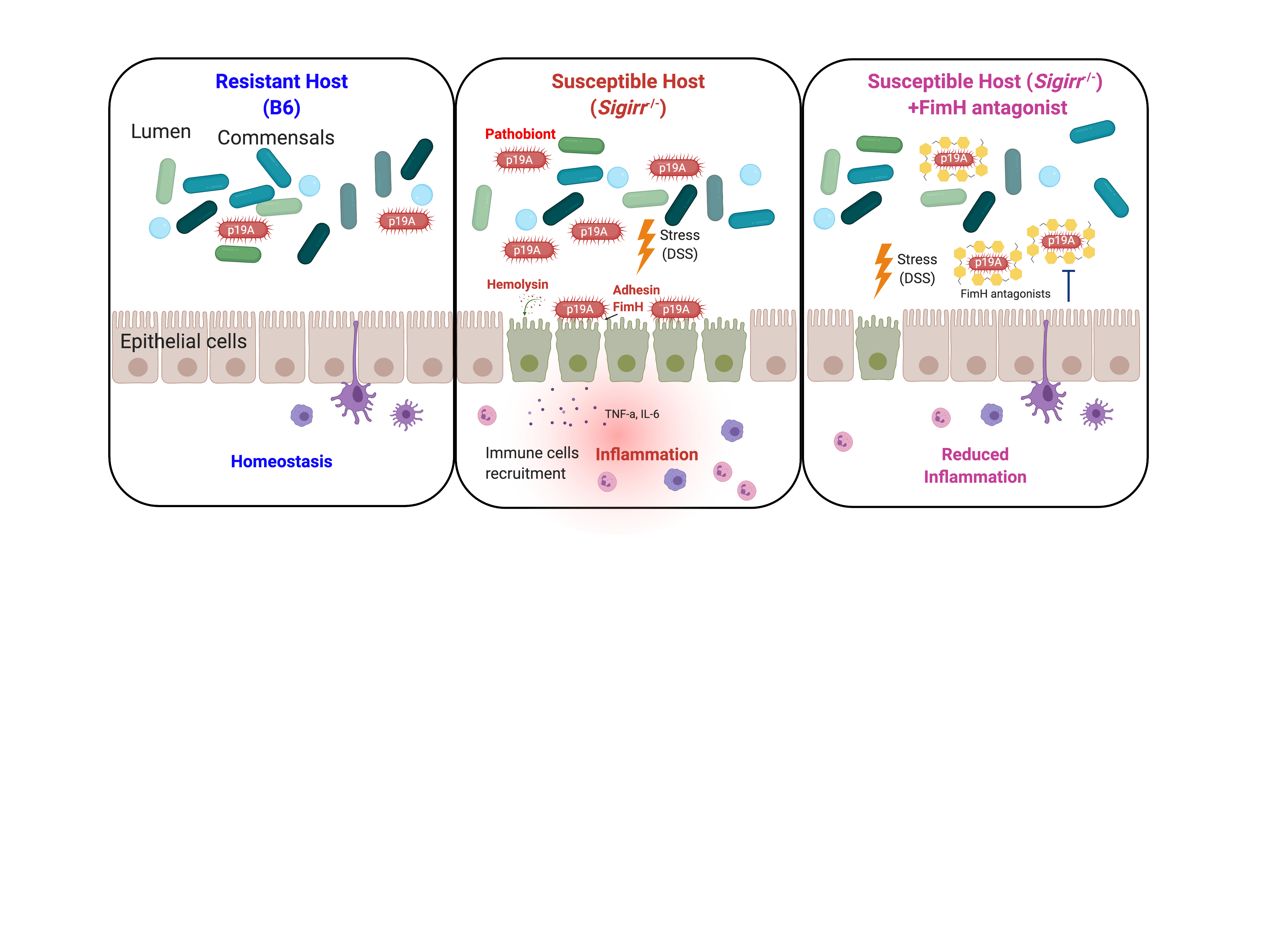

Supplement: Supplemental Material [file KGMI_A_1847976_SM9787.zip › Supplementary information/Figure S6.jpeg]
